# Spectacles of Settler Colonial Memory: Archaeological Findings from an Early Twentieth-Century “First” Settlement Pageant and Other Commemorative Terrain in New England

**DOI:** 10.1007/s10761-021-00635-2

**Published:** 2022-01-01

**Authors:** Meghan C.L. Howey, Christine M. DeLucia

**Affiliations:** 1 Anthropology Department, University of New Hampshire, 73 Main Street, Durham, NH 03824 USA; 2 Earth Systems Research Center, Institute for the Study of Earth, Oceans and Space, University of New Hampshire, Morse Hall, Durham, NH 03824 USA; 3 History Department, Williams College, Hollander Hall, Williamstown, MA 01267 USA

**Keywords:** Memory, Settler colonialism, Spectacle, New England, Replicas

## Abstract

In 1923, rural New England mill town Dover, New Hampshire, staged a Tercentenary pageant of extraordinary proportions to celebrate its “first” settlement. This public spectacle memorialized a specific, and deeply exclusionary, narrative of English settler colonialism, shaped by social anxieties of the post-First World War United States. Recent archaeological research has found possible remnants from this spectacle on a seventeenth-century site. In disturbing this site, the Tercentenary pageant appears to have disregarded actual significant material traces from the very era it aimed to memorialize--traces that offer distinct, fuller understandings of deeply nuanced Native-settler interactions in the Piscataqua River region. Dover’s pageant is situated in a regional analysis of Native and Euro-colonial commemorative place-making of the early twentieth century, exploring how different communities pursued multivocal, monovocal, or other approaches in their performative engagements with the seventeenth century.

## Introduction

*“Welcome, Welcome Columbia, and you her daughters, Fair United States, Welcome to Strafford County and the Historical Pageant of Dover.”*

**Fig. 1:**
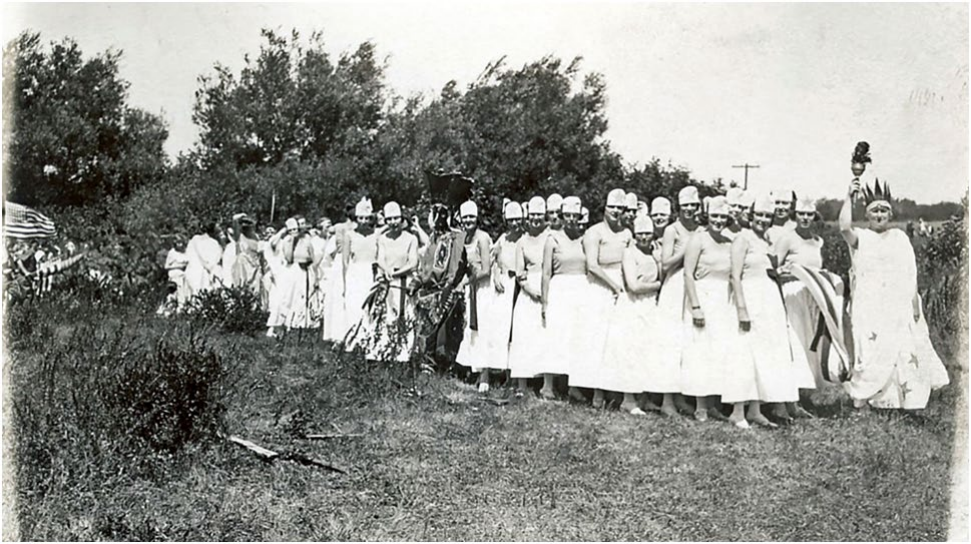
Columbia and her daughters lined up to start Dover, New Hampshire’s Tercentenary Pageant in 1923. Note the Euro-American performer dressed as a Native American. This kind of racialized form of “playing Indian,” to invoke Dakota historian Philip J. Deloria’s (1998) concept, was commonplace in this period and publicly manifested in historical commemorations (Dover Public Library: https://www.dover.nh.gov/government/cityoperations/library/historical-images/events/dovers-300th-anniversary-pageant.html)

**Fig. 2:**
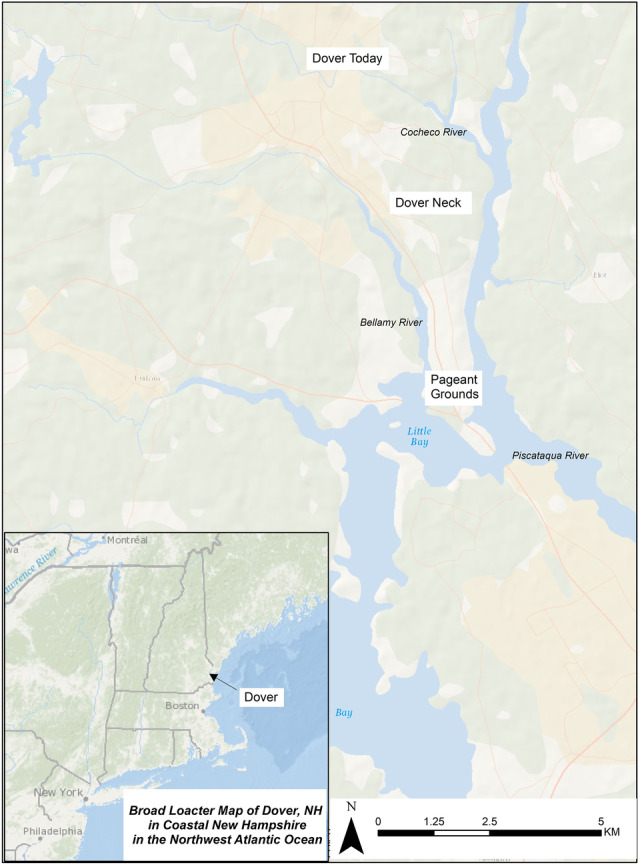
Locator map of contemporary Dover, New Hampshire, showing early colonial Dover on Dover Neck and the Pageant Grounds (Base Data ESRI)

So began the spectacle that was Dover, New Hampshire’s Tercentenary Pageant, one part of the *Tercentenary Celebration of the First Settlement of New Hampshire at Dover, August 18 to 23, 1923* (*Book of the Pageant* 1923) (Fig. 1). At 10:00 am on Tuesday August 21, 1923, over 20,000 spectators came via train, by automobile, and on foot to pageant grounds on Dover Neck, a protruding neck of land bordered by the Bellamy River to the west and the Piscataqua River, the highway to the Atlantic Ocean, to the east (Fig. 2). The town of Dover was (and still is) centralized further inland along the Cochecho River, but Dover Neck was the primary location of early English colonial settlement throughout the 1600s and early 1700s. It is on the point of this neck where the earliest English colonial “permanent settlement”-- that of fishmonger Edward Hilton, celebrated by the Tercentenary -- is argued to have been made in 1623. In this location the gathered spectators watched upwards of “1100 performers in costumes unfurled in pageantry the history of the founding of the first permanent settlement in New Hampshire at that very spot three centuries ago” (Coblenz 1923). The performance repeated on Wednesday August 22 to similar fanfare, being heralded as the highlight of the six-day celebration of Dover’s Tercentenary. The Tercentenary organizers even hired the nationally recognized John B. Rogers Producing Company (headquartered in Fostoria, Ohio) to professionally produce the pageant.

Father Time narrated the pageant, which moved through a series of historical episodes (*Book of the Pageant* 1923)*.* For many of the episodes, sets with structures were built and modified during the acting out of the performance. The first episode was a crude representation of pre-European-contact Indigenous peoples of the region called “Indian Camp and Primitive Occupations.” The pageant’s historical vignettes moved quickly to the early colonial period, and the majority of the pageant’s episodes emphasized a progressive narrative of the endurance and success of English colonizers domesticating the “wilderness” encountered here, including Native Americans. Several scenes depicted Englishmen trading with Indians, lumbering, “building of garrison houses,” and similar domestic improvements.

**Fig. 3:**
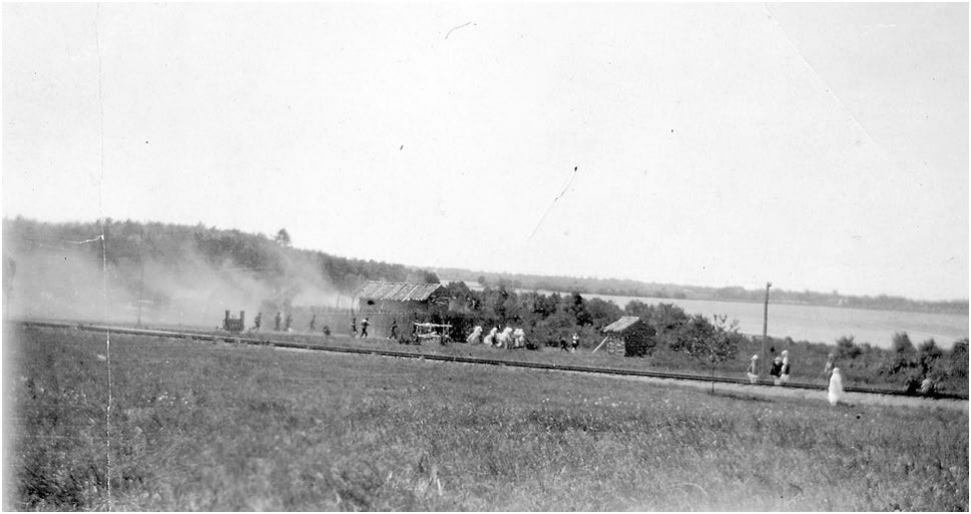
The pageant performatively reproduced the burning of colonial military and civic leader Richard Waldron’s 1600s garrison. The structure had been targeted in a 1689 “massacre”--as called by colonists--in which regional Native coalitions forcefully pushed back against colonization pressures (Dover Public Library: https://www.dover.nh.gov/government/cityoperations/library/historical-images/events/dovers-300th-anniversary-pageant.html)

A particularly contentious event, the “Destruction of Cochecho, 1689,” featured in episode six of the pageant. This highlighted an infamous “massacre” wherein Major Richard Waldron, a prominent colonial government official in Dover--who 12 years prior had tricked hundreds of Native Americans in a sham battle (performed in an earlier pageant episode) and sent many into slavery--was killed by Native Americans. The pageant predictably emphasized the formidable Natives and their aggression, not Waldron’s deceptions, nor the larger violences of settler colonialism. To emphasize this moment, performers set a mock-up of Waldron’s garrison on fire (Fig. 3). White men performed the roles of Native leaders including Wonalancet, Kancamagus, Mesandowit, and other Algonquians, while the honor of performing Richard Waldron fell to Dover’s mayor, Charles G. Waldron. He, like numerous cast members, was a descendant of the original settler families, giving an air of uncanniness to the restagings as narratives of settlement and rightful land ownership were affirmed through the bodies of blood relations who still laid claim to those benefits (one descendant-actor recollected the pageant later in life: see Smith (1973). The pageant’s historical vignettes extended through the 1788 ratification of the Constitution of the United States by Doverites. Organizers attempted to achieve factual accuracy by convening a “Committee on Historical Information” to weed out untruths, yet ultimately dramatic thrills trumped pedantic insistence on historical rigor.

This pageant was a performance of exceptional proportion for a small, rural, post-industrial mill town such as Dover in 1923, which had a population of 13,029 as reported in the 1920 census. Reckoning with this pageant as a *spectacle* offers significant opportunities for critical engagement with its meanings, both in 1923 and into the present. A spectacle is a form of theater that entails a certain size and grandeur, involves movement and action, is dynamic in form, requires spectators voluntarily in attendance, and gives primacy to visual, sensory, and symbolic codes (MacAloon 1984). Spectacles are well-suited to be events and spaces of political and social significance that create potential for communicating messages about power and hierarchy (Coben and Inomata 2006; DeMarrais 2014). These productions often involve high costs of staging the “spectacular” to make them evocative for spectators (DeMarrais 2014). Spectacles are not secondary or epiphenomenal to social processes (Inomata and Coben 2006: 32). Rather, as evocative productions, spectacles are a “public display of a society’s central meaningful elements” (Beeman 1993:380). In the “veritable passion for historical pageants [that] persisted through the interwar years,” American historian Michael Kammen (1991:422-423) has written, theatricality, commercialism, and civic promotional interests converged as communities used these enactments of selective, often highly nostalgic and romanticized, versions of the past to inculcate identity and meaning in the present (see also Glassberg 1990; Phillips 2021). The Dover Tercentenary Pageant marshalled local Euro-colonial residents’ minds, bodies, and resources to develop spectacular performative relationships with the past.

In the analysis that follows, we contextualize the rise of interest in publicly, visibly celebrating early settler colonial history in New England. Many factors converged in 1923 in this mill town, and it is important to situate the Dover Pageant in particular localized conditions of the post-World War I United States. This context then provides framing for an interpretation of recent archaeological findings as possible evidence of the enactment of this spectacle, which aspired to memorialize a specific--and starkly exclusionary--narrative of early English settler colonialism. In doing so it may have disregarded significant material traces from that era that offer distinct, fuller understandings of actual geographies and lifeways in the seventeenth century, including nuanced Native-settler interactions and entanglements. Finally, we situate the Dover Pageant within a regional analysis of Native and Euro-colonial commemorative place-making of the early twentieth century, exploring how different communities pursued multivocal, monovocal, or other approaches in their performative engagements with the seventeenth century. This comparative perspective leads us to assess the shifting importance of material “replicas,” and the possibilities for alternate forms of remembrance in the 21st century.

## Trajectories of Collective Remembrance of Early Colonial History

Prevalent Euro-colonial narratives of New England’s origins have long valorized an image of the region as arising from innocent, morally upright ambitions and actions carried out by Anglo-colonial arrivals. These mythologies have typically downplayed or disavowed the enormous contestation and violence entailed in the colonization project, including the genocide of Native peoples, attempted dispossession of politically sovereign Native nations from traditional homelands, and imposition of racial slavery that severely impacted the lives and bodies of Native as well as African American peoples subjected to regimes of unfreedom (Hardesty 2019; Matthews and McGovern 2015:2; O’Brien 2010; Warren 2016). When violences have been acknowledged in such narratives, their inclusion has often served to reaffirm Euro-colonial identities as borne out of existential struggle to overcome obstacles to settler colonial expansion, social and capitalistic flourishing, and political liberty. These trends have shaped colonial meaning-making in the Piscataqua region and northern New England more generally, as well as in the parts of southern New England that have attracted considerably more attention from scholars in history, archaeology, literature, and related disciplines.

(Mis)representations of New England histories are politically forceful projects in which erasures and silences are not neutral. As many Native and African American communities in and beyond the region have long known through lived experience and intergenerational memories as well as community-based research, and as scholars are increasingly beginning to recognize, such representations are consequential for whose realities are recognized, legitimized, and valued in the present day; and for how communities perceive connections between past and present--or deny them. As Michel-Rolph Trouillot (1995) emphasized in *Silencing the Past*, the construction of historical narratives and social memories is closely bound up in the exercise of political power and the ability to assert specific truth-claims. Some truth-claims reinscribe and naturalize relations of inequity and domination in the present day. Others seek to resist, subvert, and transform them.

Native people in the Piscataqua region have maintained memorial and knowledge-keeping traditions since time out of mind through multigenerational oral traditions, place-based relations, material culture practices, and, beginning in the seventeenth century, strategic uses of writing in documentary forms. This is essential Indigenous context--offered very briefly here--in which to situate the *colonial* forms of historical representation that have strongly shaped the Dover milieu. In New Hampshire the first concerted effort to document early colonial history using English-language writing was by Reverend Jeremy Belknap, who lived on Silver Street in Dover while composing *The History of New-Hampshire* (1784-92). This book remained a landmark account of the New Hampshire past for more than a century. *The History of New-Hampshire* emerged in a moment of surging interest in the early American past and fascination with local heritage, or what scholar Alea Henle (2020) has referred to as “historical cultures”-- formal, often institutionalized, modes of preserving and interpreting the past, frequently in ways that rationalized and honored the growth of the newly constituted US nation. The Massachusetts Historical Society (established 1791) embodied this antiquarianism: it counted Belknap among its founders, giving Belknap and his work a notable place in the emerging field of American antiquarianism (Tucker 1990). The Historical Society of New Hampshire emerged a generation or two later, being founded on May 21, 1823. In choosing the bicentennial of English colonial settlement to establish a state historical society, founding members deliberately invoked 1623 as a key “beginning.” Beyond formal institutions, settler memories of early colonial New Hampshire also became articulated in popular media such as poems, plays, and novels (Jewett 1896; Wheeler 1881; Whittier 1868).

Even within a small state like New Hampshire, significant local variation existed in how Euro-colonial communities approached heritage and memorialization. Interest in marking historical landscapes and collectively remembering early colonial history was always more pronounced in the relatively more affluent, urban, coastal town of Portsmouth than in Dover, the latter being not only more inland but also more of a working-class mill town. Whereas Portsmouth boasted institutions like the Portsmouth Athenaeum (est. 1817), a New England type of salon modeled after the Boston Athenaeum (Porter 2002), in Dover residents and avocational historians had more diffuse interests and points of social connection. Toward the end of the nineteenth century, prominent town residents, like Dover town librarian Caroline Harwood Garland, worried about how little Dover had done to organize cultural institutions around its rich early historic materials. Garland expressed anxiety, shared by others, that without formalized memorialization and celebration of early colonial history, Dover’s old New Hampshire stock--meaning Yankees, descendants from original English colonists--would become too intermixed with new immigrants coming to Dover to work in the mills, and would risk losing their uniqueness and identity (Garland 1897: 113). As these nativist anxieties about ethnic diversity grew, certain Euro-colonial residents in Dover worked to institutionalize collective forms of remembrance through a succession of historically minded organizations and social groups, loosely focused on celebratory forms of so-called “colonial revival” (for a survey of colonial revival in the broader region, see Giffen and Murphy 2004).

The Dover Historical Society organized in 1889 (see Dover Historical Society 1891) and its members did begin to compile town records, but the group never became especially active. Another antiquarian presence developed in Dover around the same time: the Northam Colonists, an all-volunteer organization that organized in 1895 and officially incorporated on September 1, 1900 (*Northam Colonists Papers*). The group’s named derived from the fact that Dover went through a series of names in its early colonial period, one being Northam, which remained in use for a few years in the late 1630s until the region was sold back to Massachusetts and renamed Dover in 1641 (Scales 2008 [1923a]). Membership in the Northam Colonists was restricted, requiring genealogical proof of descent from the original English settler colonists of Dover, starting with Edward Hilton. The Northam Colonists represented the major antiquarian presence in Dover for decades and devoted their meetings and activities to presentations and papers on colonial topics.

These “heritage” interests attained physical expression in public spaces. The most visible addition to the antiquarian landscape was the Woodman Institute Museum. Dover resident Annie E. Woodman, a local philanthropist who had been married to the late banker Charles Woodman, bequeathed $100,000 “for the establishment in Dover of an institution for the promotion of Education in Science and Art and the increase and dissemination of general and especially historical knowledge” (Annie E. Woodman Institute 1916:3). Woodman was part of a broader trend of educated Euro-colonial women leading historic preservation efforts across the country (see Matthews 2012). Her gift helped establish the Woodman museum in 1916 as a private museum that would showcase “the early history of Dover and of New Hampshire,” and be “a constant and active factor in the intellectual life of Dover” (Annie E. Woodman Institute 1916:16). Seeking to offer historical authenticity to the operation, and to focus settler memory around a tangible structure, museum trustees hired a local contractor in 1915 to move an early colonial “garrison” house dating to the 1600s 3 mi (4.8 km) to the museum. In its new location the house became a centerpiece for visiting and interpretation, and it still stands today as a site of early colonial settler memory (DeLucia 2012).

### Settler colonial memory in post-World War I Dover, NH

The year 1923 marked a banner year for celebrations of early colonial settler heritage as Dover, as well as other communities throughout the Piscataqua region, formally commemorated the tercentenary of colonial settlement. These events responded to changing social, political, and demographic conditions in the region as well as the nation. In the years prior to the First World War (1914-18), immigration to the United States grew substantially: before the conflict, over one-third of US residents had been either born abroad or were the offspring of parents who had immigrated to America (Reft 2017). Many of these immigrant residents labored in places like the mills of Dover, where in the early 1900s, half of the mill workers were French-Canadian immigrants, 13% were Irish immigrants, and 10% were Greek immigrants (Beaudoin 2002).

While nativism and xenophobia certainly existed in the region prior to this era, the war fostered particular forms of nationalism and civic identity-formation that exacerbated Anglo-colonial antagonisms and anxieties about losing “Yankee” footholds (Kennedy 2004: 24). A global conflict fought on multiple continents, the war and its associated military innovations--and high levels of destruction and casualty rates--helped make, unmake, and remake individuals, cities, nations, and continents in unprecedented ways (Saunders 2003: 1). To galvanize hesitant Americans into entering the war, which happened in the spring of 1917, US President Woodrow Wilson’s administration pushed an aggressive propaganda campaign that emphasized Americanism (Wood 2014: 277; also see Creel 1920). Americanism promoted a vision of citizenship as rooted in shared values, dramatically revising American notions of civic belonging and cultural difference (Blakey 1970; Capozzola 2008; Kennedy 2004; Price and Howey 2016).

Immigrants served in the military at disproportionately high rates, and also labored in wartime industries in significant numbers (Reft 2017). Despite this wartime service, the longing for American unity fostered by war propaganda and efforts raised a specter of unassimilated immigrants seen as threatening US security and futures, and fostered growing anti-immigrant sentiments (Capozzola 2008; Kennedy 2004; NPS 2021). After the war, the United States increasingly trended towards nativist, nationalist views and promoted the rapid assimilation of immigrants already living within US borders. The US also began closing its “open door” immigration policy, and the Immigration Act of 1924 established the restrictive visa system still used today (NPS 2021).

The Dover Tercentenary Pageant reflected this broader nationalist milieu. Lauding a dramatic and progressive narrative of settler colonial success--defined in ways that explicitly excluded certain spectators at the event--reinforced Anglo-colonial descendants’ claims to occupy the top-tier position in a hierarchy of citizenship, and promoted overall imperatives for homogeneity in citizenship. Given that spectacle can be considered a “public display of a society’s central meaningful elements” (Beeman 1993:380), this pageant can be viewed as publicly displaying a particular type of meaning: that in Dover, *true* affiliation and belonging were the domain of people who descended from the town’s original English colonizers. They were, as the pageant materials expressed it, the “inhabitants of this goodly town” who had “ancient” connections to this place, unlike recent, non-western European immigrants (*Book of the Pageant* 1923)*.* In privileging purist claims of Anglo-colonial descent, the English descendent actors who claimed prime roles staked out clear lines of priority and authenticity linking *certain* citizens from the past with the present.

In the pre-performance welcome, pageant spectators received a “cordial welcome” that remarked, in part, upon whether they “were born within its [Dover’s] ancient boundaries, interlacing your affection with those with whom you compose this incorporated brotherhood in the great family of the union, or whether you have forsaken your home in some foreign country, renouncing all allegiance unto it” (*Book of the Pageant* 1923). The forceful word choices in the latter clause--forsaken, renouncing--emphasized that more recent immigrants were only conditionally welcomed into this space and civic body, contingent upon their willingness to assimilate, become homogenized with Dover’s “incorporated brotherhood,” and relinquish particular ethnic or national affinities with their places of origin. To underscore this vision of citizenship, the whole performance concluded with “The Melting Pot of the Nations,” a tableaux in which “peoples from every corner of the earth, unite and are amalgamated into one and an inseparable nation.”

By describing English colonists as having “ancient” roots in the area, this staging also contributed to the erasure of the reality of Native Americans’ multi-millennia ancestral connections and presences in this very land (Handsman 2008). The much longer time-horizon of Indigenous Algonquian homelands and communities became eclipsed by the three-century English colonial presence. And while the pageant featured “playing Indian” in its early scenes, it kept Native presences (albeit through the lens of racialized and caricatured figures) relegated firmly to a historic past, rather than recognized as ongoing peoples and presences in the twentieth century. Yet it is certainly possible that Native people attended this very spectacle. As census and other documents demonstrate, Native people from multiple tribal communities were indeed resident in the greater Piscataqua region in this era, reflecting complex experiences of displacement and migration, employment-seeking, and network-building (DeLucia 2012). While documentary records about the composition of the pageant audiences in Dover are limited, it is apparent from other Northeastern contexts that Native people regularly witnessed commemorative activities--and sometimes pointedly delivered counter-narratives that disproved Euro-colonial claims about Indigenous “vanishing,” using their very embodied presences to publicly mark Indigenous continuance and modernity (for accounts of this pushback in Wampanoag and Narragansett contexts, see Blee and O’Brien 2019; Rubertone 2020).

### Archaeological Signature of Enacting the Tercentenary Pageant Spectacle

In crafting a spectacle to publicly reinforce Anglocentric narratives and hierarchies of citizenship and belonging, the organizers of Dover’s Tercentenary praised the fact that they were performing precisely where the earliest English settler colonists had been located. Local historian John Scales (1923b) remarked in a newspaper account published before the event began that it had been his privilege to “visit the site selected for the presentation of the pageant, on Dover Neck.” He wanted spectators to know that they would be sitting on the *very spot* where the historical events they were watching “were transacted.” He urged spectators to recognize that when they “enter that exhibition field they are treading on history.” The conceit of “treading on history” signals the values that assertions of locational authenticity held for pageant organizers and attendees, as well as the complexities presented by engaging with material traces in these locales.

**Fig. 4:**
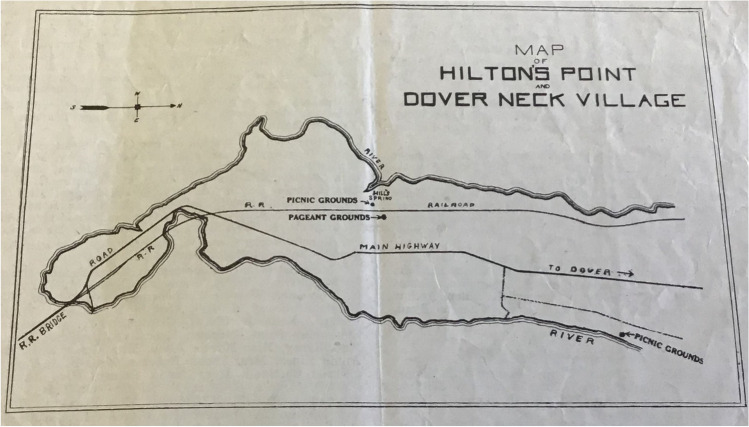
Map of Tercentenary Pageant grounds published in *Foster’s Daily Democrat* (August 1923; Dover Public Library Special Collections, image by Diane Fiske, GBAS community historian)

Figure 2, above, presents a broad locator map of the pageant grounds, while here Fig. 4 shows an August 1923 published map of the pageant grounds and associated picnic grounds on Dover Neck. Since the main economic activities pursued by very earliest colonists in the area in the 1620s and 30s were fishing and trading rather than agriculture, early (and only male) colonists received grants of 20 ac (8.1 ha), which established a compact community on Dover Neck, often called Dover Neck Village in historical records. These grants arose within larger contexts of settler colonialism in Native Northeastern homelands in the seventeenth century, and amid Native leaders’ strategic efforts in the Piscataqua region to negotiate rights and responsibilities with English colonizers, who were keen to secure what they envisioned as legal title to these valuable lands (Baker and Maurer 2018). As Fig. 4 helps illustrate, pageant organizers purposefully sited the event as close as possible to the center of these early colonial settlements.

This geographic strategy--in which *authenticity of place* amplified the colonial claims to Dover--complemented the emphasis on purity of English identity reckoned through genealogical descent. As scholars of place and memory have noted (e.g., Rubertone 2008a), landscapes are multilayered, multivalent spaces in which diverse communities frequently derive meaning and identity from interaction with sites of past significance. The memorial “placemaking” in which the pageant organizers were involved demonstrated high attunement to the terrain in which Anglo-colonial ancestors transformed space through their labor as well as affective ties. This colonialist form of placemaking prioritized the salience and value of *English* historic home-places, ignoring or erasing the complex Native homelands terrain that in fact far exceeded the former in geographic extent and temporal depth.

### Dover’s Early Colonial Meetinghouses

**Fig. 5:**
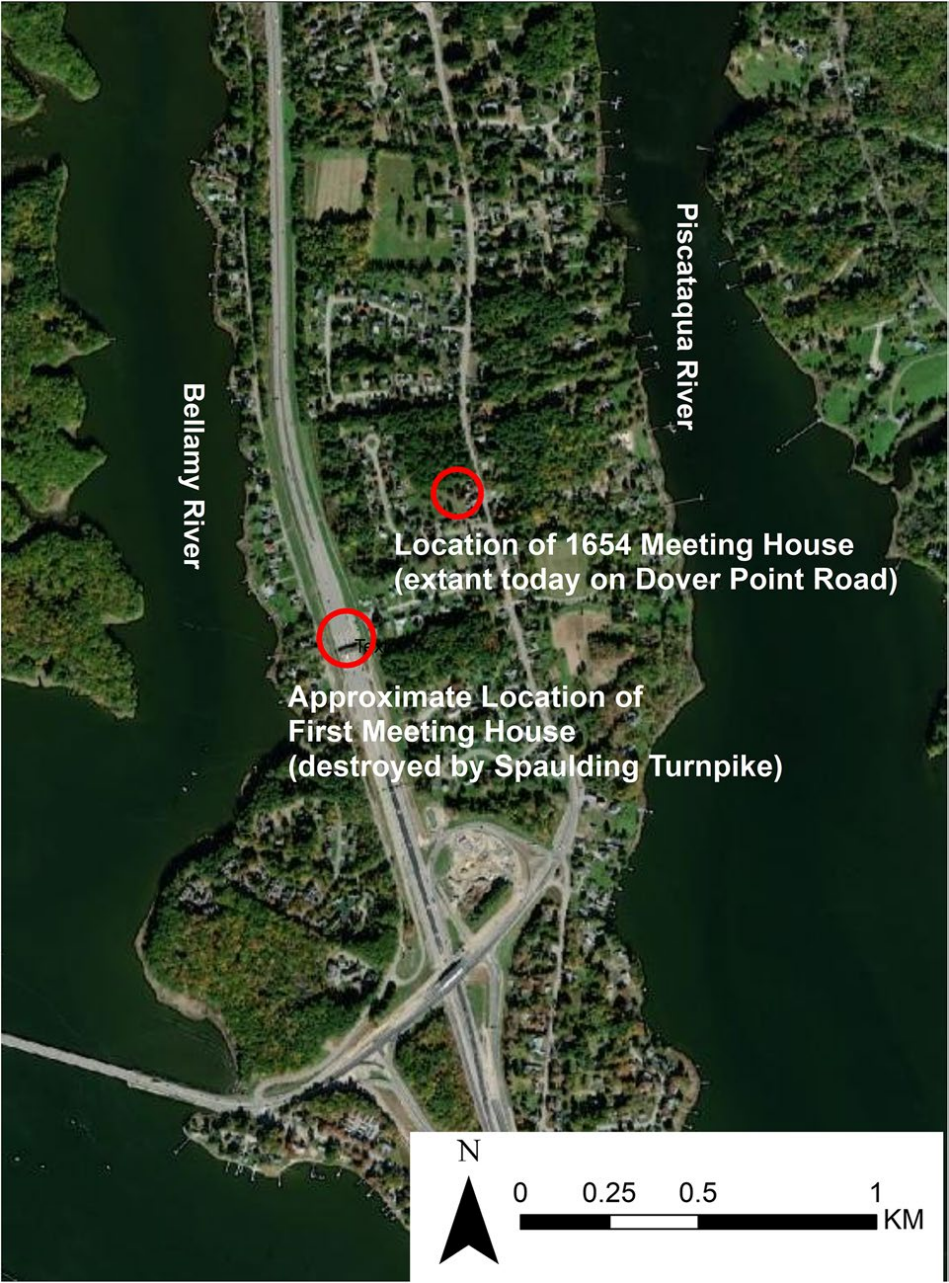
Location of First Meeting House (center of Pageant grounds) and the nearby second meeting house built in 1654, still extant today and the focus of GBAS field work (base data from ESRI).

An essential element of the early colonial built environment on Dover Neck was its meetinghouse. English colonists erected the first meetinghouse near Hill’s Spring in 1633 (Figure 4 and 5). Reverend William Leveridge preached the first sermon at the meetinghouse on October 30, 1633, a date subsequently recognized as constituting the beginning of the First Parish, or civic community (Quint 1877). At that time, the town and the parish were one and the same, and civic and religious life operated in the same structure. That meetinghouse served as the town meeting house, courthouse, and house of worship. For more than a century, colonial identity actively took shape through residents’ participation in worship here, since all colonial inhabitants of the area were required to attend this meetinghouse for worship (Dover was not formally pluralistic in its religion: Quakerism, for instance, was not officially tolerated). Not until 1762 did the First Church become incorporated as a Parish distinct from the town of Dover (Wadleigh 1913: 151). This first meetinghouse site attained enormous prominence in the 1923 pageant, with its location harboring the pageant ground (Scales 1923a).

As the English colonizing population at “Dover” or “Dover Neck” increased by the mid-seventeenth century, it outgrew this initial meetinghouse. Colonial leader Major Richard Waldron agreed on August 8, 1652, to construct a new meeting house on Nutter’s Hill located upland from the first meetinghouse, less than 1 km (0.6 mi) to the northeast (Fig. 5). This second meetinghouse location was on the main terrestrial travel route that eventually became the first major street in the area, called variously High Street, Main Highway, and today Dover Point Road. Early colonial records specified the material form of the structure:

Mr. Richard Walderne doth bind himself to erect a meeting house on the hill near Elder Nutter’s; the dementions of said house is to be forty foot longe, twenty six foot wide, sixteen foot stud, with six windows, two doors, and to plancke all the walls; with glass and nails for it; and to be finished betwixt theis and April next, come twelve months, whc will be in the year 1654. [original spellings; Dover, NH Town Records 1657-1753; see also Wadleigh 1913:35]

It appears that the meeting house was not completed in 1654, as shown in another town meeting record in 1658 (*Dover, NH Town Records* 1657-1753). Here, town residents voted that the meeting house be underpinned, catted, and sealed with boards, and a pulpit and seats made. In today’s terms, that means that it was to be propped up, or the foundation strengthened, and the finish work inside completed, as well as adding seats and a pulpit. Colonial residents also voted at this time to purchase a bell. Two years elapsed in completing these repairs, as shown in Old Dover Records in June of 1660, and a tax of 100 pounds was voted to be assessed to fund the capital improvements (*Dover, NH Town Records* 1657-1753). In 1665 town officials gave directives for a turret to be built on the meeting house in which to hang the bell that was brought over from England by Waldron (*Dover, NH Town Records* 1657-1753). The prominence of the bell and enclosing tower in these plans demonstrates the sonic dimensions of affirming and regulating colonial civic identity. Its tolling--audible to anyone in the vicinity, whether colonizer or Native--would have been a sensory reminder of the lands and power claimed by English colonizers.

**Fig. 6:**
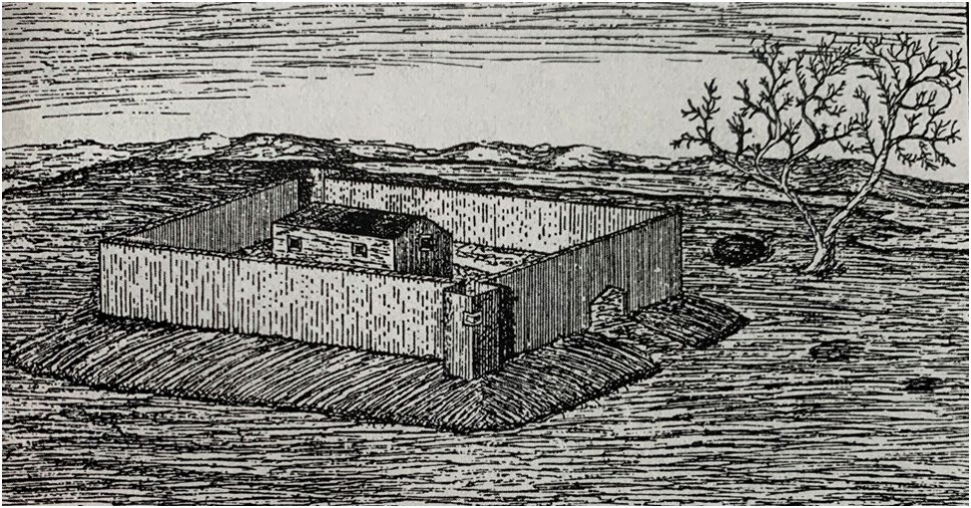
The only image presently known to show how the fortified second meetinghouse on Dover Neck may have looked. It was published in the front of George Wadleigh’s (1913) “Notable events in the history of Dover, New Hampshire from the first settlement in 1623 to 1865.” No artist credit was given, and it was labeled only as “The Old Meeting House on Dover Neck.”

The architectural evolution of this meeting house illuminates the complex dynamics of colonial-Indigenous relationships in this area, which was simultaneously a longstanding Abenaki and Pennacook homeland and an English “frontier.” In 1667 the Massachusetts General Court ordered that a stockade fence be erected around the meeting house, forming a 100-ft (30.5 m) square with 8 ft- (2.4 m) high walls of 12 in- (30.5 cm) thick timber, also comprising sills and braces, and two watchtowers 16 ft (4.9 m) square (Fig. 6). Colonial residents of Dover pushed back against this requirement, which likely would have entailed significant expenses and labor. Their reluctance to construct the specified fortification indicates that most English colonists in the area did not at that time experience an urgent sense of threat from local Indigenous people, nor from competing colonial polities like the French to the north. The political stronghold of colonial New England was south of Dover, centered in Boston. For the early decades of colonial presence in Dover the region was loosely governed and these “frontier” colonists were largely focused on individual pursuits of economic gain through fishing and lumbering (Baker and Mauer 2018). By the 1640s, however, the Massachusetts Bay Colony had taken official control of Dover and began exerting more influence. But this area’s outlying northern location posed challenges to Massachusetts government control, and the region’s English residents routinely tangled with the Massachusetts Bay colonial government (Nelson 2019).

When the order came in 1667 from Massachusetts Bay to fortify the Dover meetinghouse, violent, hostile relationships between aggressively expansionist English colonizers and Indigenous peoples seeking to maintain their homelands and sovereignties were endemic to southern New England (for more on the dynamics of early settler colonialism, violence, and resistance in southern New England, see Brooks 2018; DeLucia 2018). However, an ongoing archaeological survey program across the region where Dover sits, and which led to the findings discussed below, has found material evidence of the non-violent presence of Native peoples at multiple seventeenth-century English sites (these findings are the subject of other in-preparation publications). Indeed, the on-the-ground realities of Native and colonial relationships in the Dover region indicate high degrees of cross-cultural interaction, trading relationships, and interdependencies. These complexities reflect specific regional dynamics. Yet the Massachusetts Court responded that if Dover colonists did not build the requisite fortifications around their meetinghouse, they would be fined and taken to court and so they were erected (Wadleigh 1913).

**Fig. 7:**
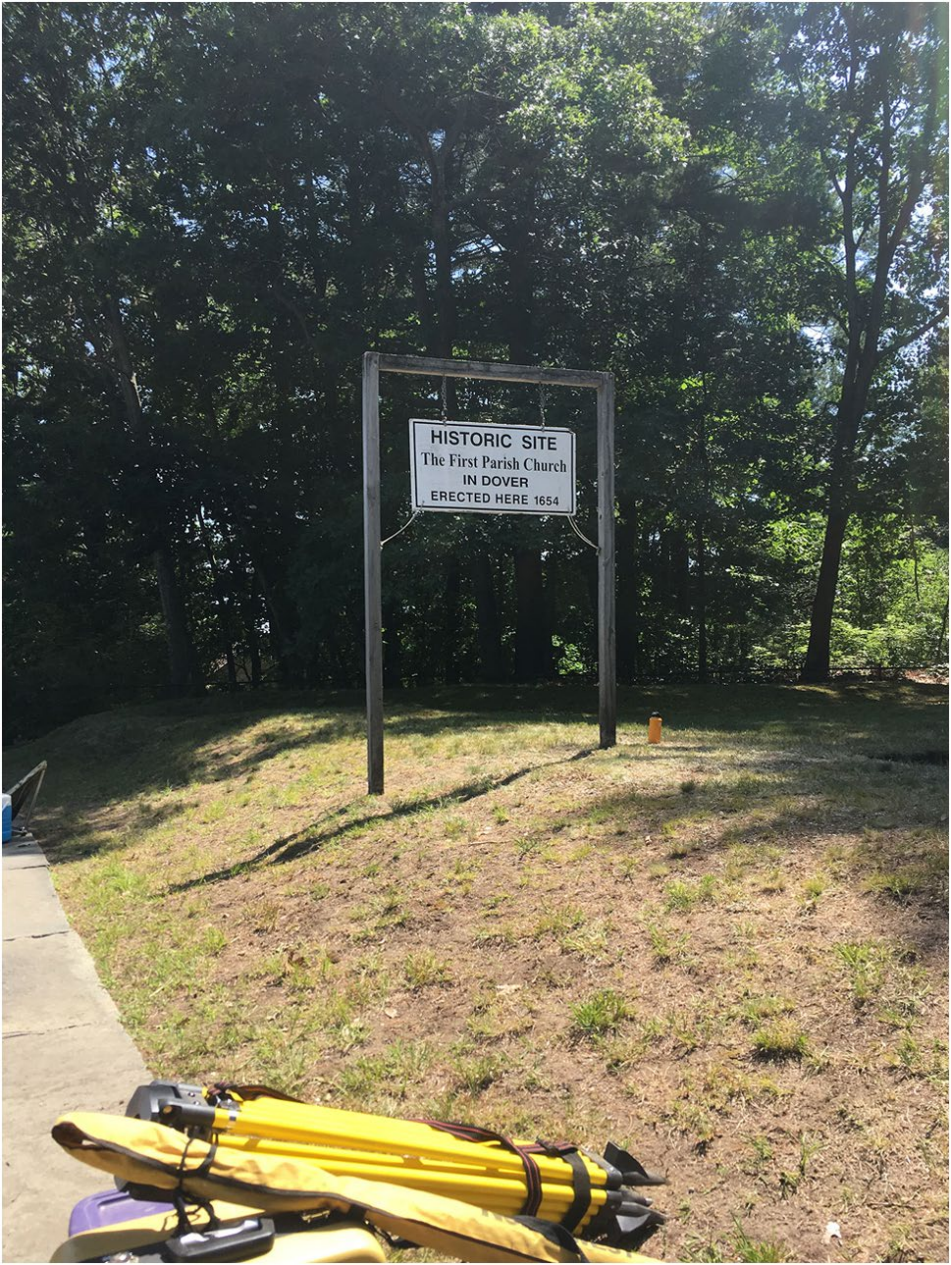
The small wooden sign marking the 1654 meetinghouse site. This photo was taken facing south at the start of GBAS fieldwork program in 2018. The earthen fortification berm is visible as is the southeast watchtower berm (photo by M. Howey, GBAS)

Only a few other records attest to repairs made to this meeting house, which appears to have been a sturdy structure. All town and church affairs were carried on in this space until 1713 when a new meeting house was built at Pine Hill, further inland from the point. Pine Hill was known as “Cochecho” and was where Dover’s center later shifted. The last recorded use of the 1654 meeting house was for a proprietors’ meeting in 1722; after that it fell into disrepair and was eventually cleared away around 1775, just before the onset of the American Revolutionary War (*Dover, NH Town Records* 1657-1753). While the building was dragged away (and its materials potentially recycled into other structures), the earthen berms built to support the stockade and watch towers remain intact in the twenty-first century. As far as available records indicate, the only other official activity that occurred on this site after the clearance was the installation in 1908 of a stone wall and iron fence by the Daughters of the American Revolution (DAR.), a heritage organization that engaged in many regional and national activities focused on colonial origins and genealogical descent (Margery Sullivan Chapter D.A.R. 1908:55). The site attained National Register of Historic Places listing in 1983 (Bryant and Starbuck 1983), and today is owned by First Parish Church. Appearing in the twenty-first century as a grass-covered lot with one large pine tree in the middle, the site has its historical significance physically and visibly designated only by a small wooden sign. Given that Dover Point Road is a busy thoroughfare, this uniquely intact, fortified early colonial meeting house site is easy to pass by without notice (Fig. 7).

### Great Bay Archaeological Survey (GBAS) Fieldwork at 1654 First Parish Meetinghouse

In 2018, the Great Bay Archaeological Survey (GBAS), of which the first author is PI, launched a field reconnaissance program at the 1654 First Parish Meeting House site (location shown in Fig. 5). GBAS is an interdisciplinary, community-engaged research program focused on surveying and excavating seventeenth and early eighteenth-century residences (e.g., garrisons and homesteads), resource extraction sites (e.g., sawmills), and civic centers (e.g., meeting houses) across the Great Bay Estuary, of which Dover, New Hampshire is part. The project’s aim is to better understand the impacts of early New England colonialism and how current environments, both natural and human, have been shaped by decisions made centuries ago during the socioecological “shock” of global colonialism. Project members are amassing a systematic collection of early colonial period (ca. 1600-1750) artifacts, ecofacts, and geospatial data and conducting material, archival, and interdisciplinary analyses of these data.

Field research questions for the 1654 First Parish Meetinghouse site included: (1) could any structural remnants of the meetinghouse be found to understand how it was constructed, repaired, and used; (2) what is the artifactual signature of an early colonial civic center and how does it differ from residential sites; (3) is there evidence of contemporaneous Native American presence at the meeting house; and (4) how was the fortification actually built? Given that the site is protected, the project team decided to approach the fieldwork in a limited way, prioritizing targeted excavations that would reveal the most information possible as related to the defined research goals.

**Fig. 8:**
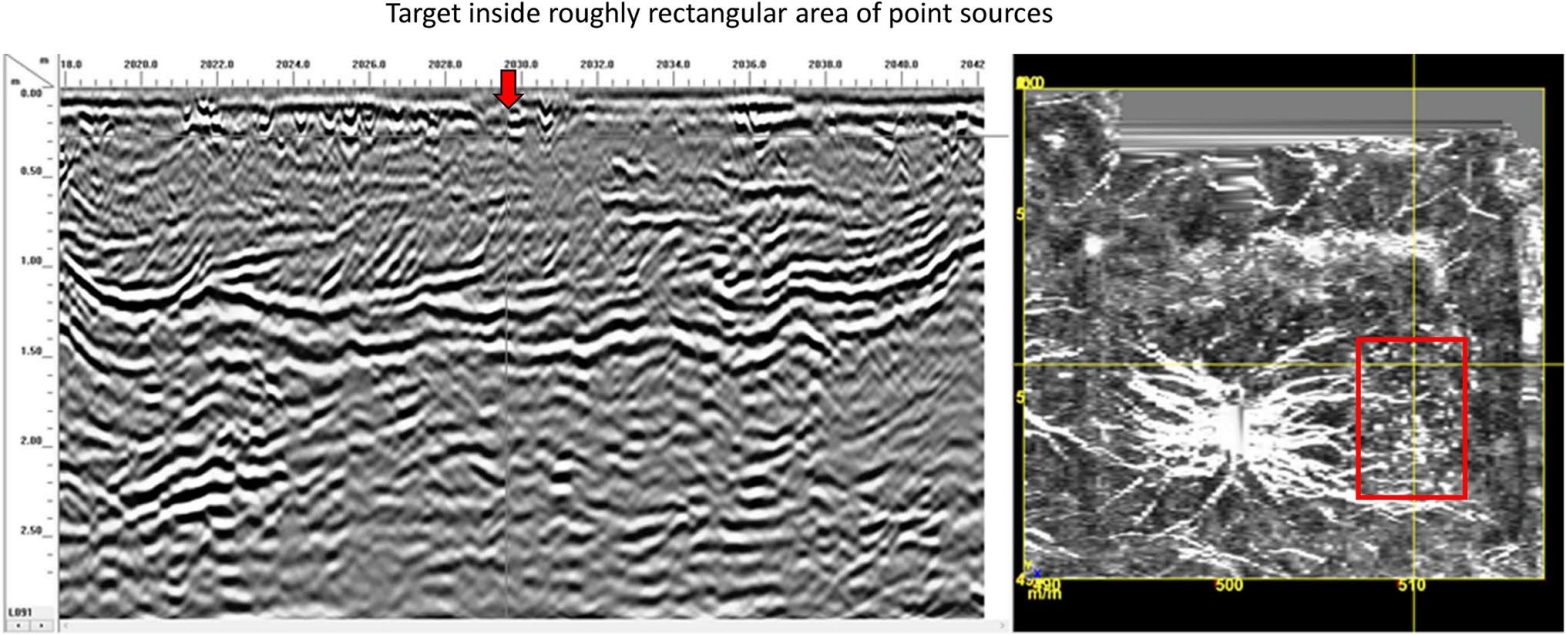
The anomaly found in GPR survey consisting of point sources in a roughly rectangular shape measuring around 8 x 12 m (or 26 x 40 ft, the reported dimensions of the 1654 meeting house). This image is the working image provided to GBAS by Peter Leech for field targeted excavation units (a full report on GPR is the subject of another working publication).

Investigation began with the non-invasive approach of Ground Penetrating Radar (GPR), an active geophysical sensing technique that uses wide-band electromagnetic pulses to produce high-resolution images of the subsurface (Leach 2021:3). A site grid was established using a total station. The site datum N500 E500 was placed close to the large pine tree within the site and the grid was laid on magnetic north. Peter Leach of GSSI ran the GPR survey at 1-m (3.2 ft) transects and to a depth of 2.5 m (8.2 ft) covering the entire area enclosed by extant earthen berms as well as across the berms, including the berms of the two watch towers. Leach conducted all post-processing. As Fig. 8 shows, the dominant signature in the GPR results is the root system of the large pine tree. Even with this disturbance, Leach provided the GBAS team six target anomalies as possible cultural features. The GBAS project team, using the total station, laid in fine-tuned 1x1 m excavation units in each of the six target locations.

**Fig. 9:**
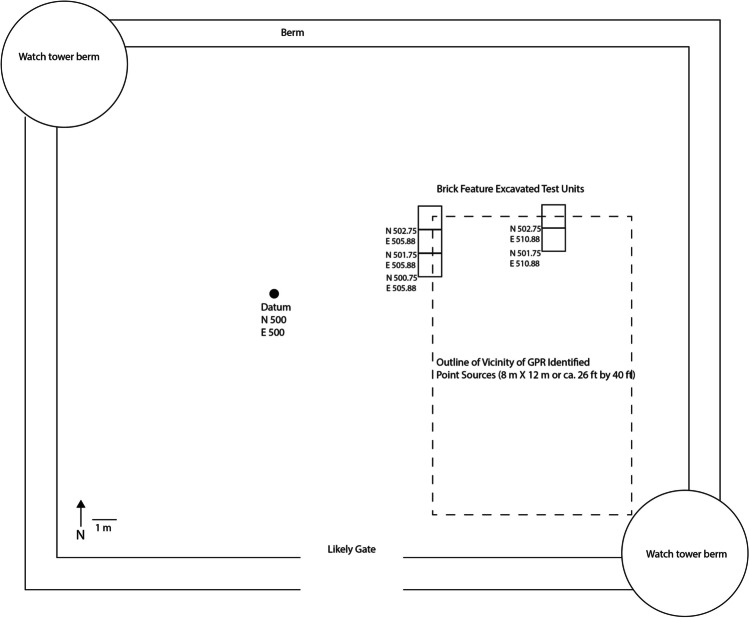
Schematic of site, location of datum, vicinity of GPR-identified point sources, and test units around this feature

One of the six anomalies identified as possibly archaeologically significant in the GPR survey was a roughly rectangular area of point sources (Fig. 8). The identified rectangular area measured roughly 8 m by 12 m (26 ft by 40 ft). These are, as described above, the historically reported dimensions of the 1654 meeting house, and so initial thoughts considered the possibility that this anomaly was the location of the seventeenth-century meeting house. The project team’s test excavations of this anomaly quickly refuted that idea but opened other questions. Fig. 9 provides a schematic of the site, site datum, this GPR-identified feature, and test excavations around it, which we now discuss.

**Fig. 10:**
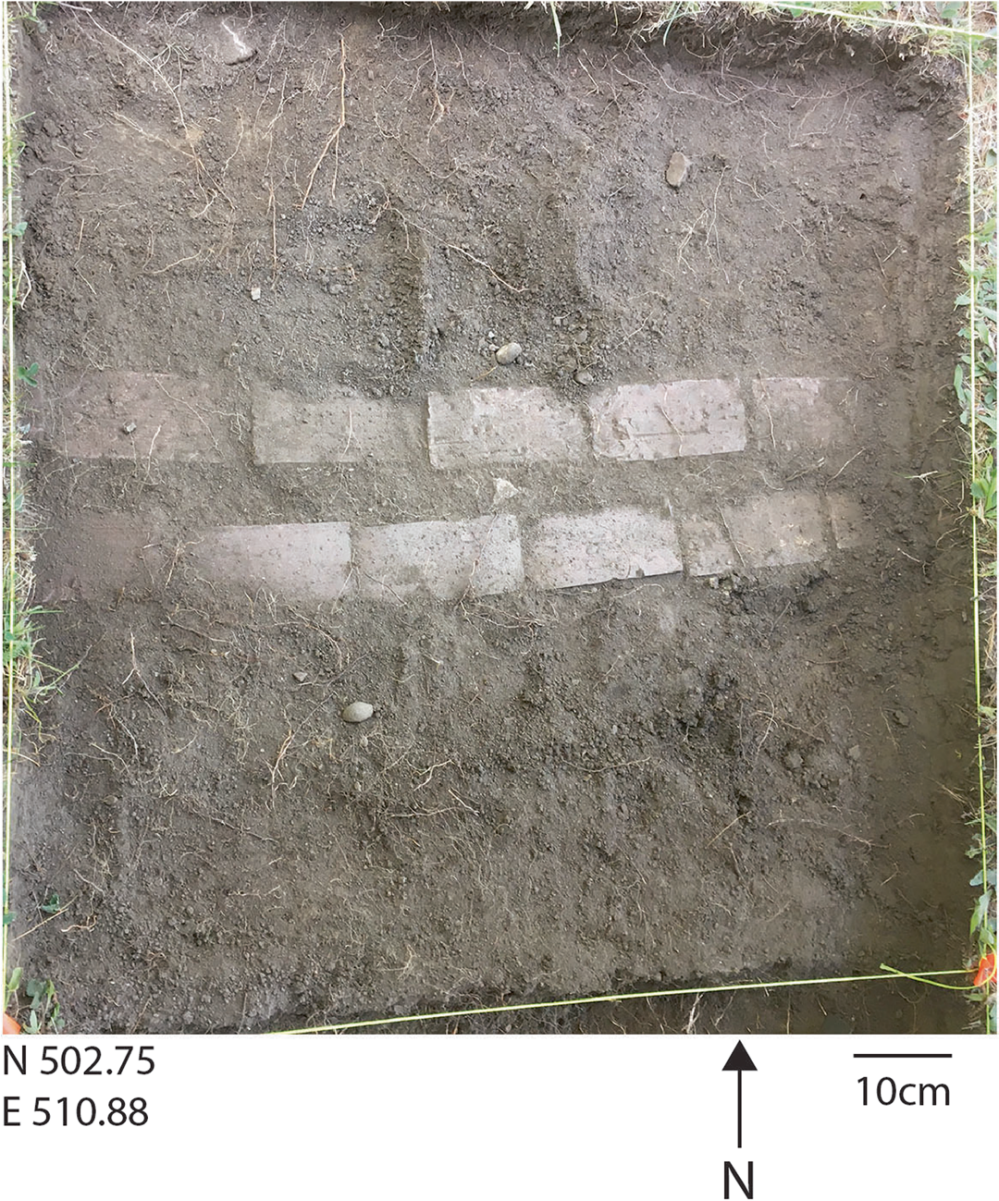
The two-by-two row of bricks encountered in the targeted 1x1 m unit N 502.75 E 510.88 during excavation of Level 1 at 8 cm below the surface (photo by M. Howey, GBAS).

**Fig. 11:**
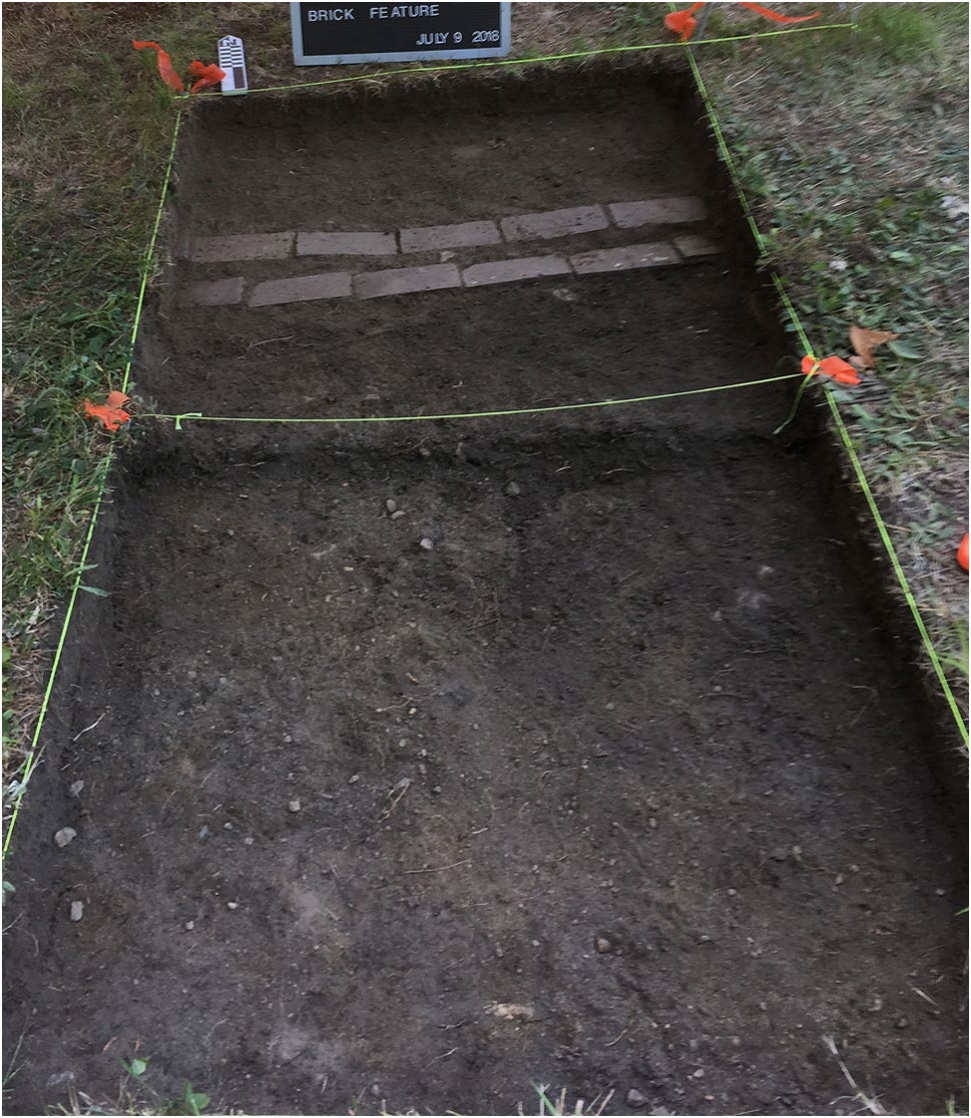
A field photo of both the original excavation unit in which the brick feature was encountered and the unit south in which no brick was encountered (N 501.75 E 510.88 and N 502.75 E 510.88) (photo by M. Howey, GBAS)

Based on the GPR results, a 1x1 m test unit was placed at N 502.75 E 510.88 (the yellow cross hairs in Fig. 8). In Level 1, at 8 cm below the surface, a row of bricks laid two by two, and oriented east to west, was encountered, shown in Fig. 10. Also evident in Fig. 10 is that the spacing between the bricks was consistent across the length of the unit, measuring just about 5.5 cm. The bricks were what caused the point sources found in the GPR (Fig. 8). Excavation was stopped and the bricks were left in situ. The project team proceeded to open the unit to the south of these bricks to assess if additional bricks were present in this unit (N 501.75 E 510.88) (Fig. 9). Bricks were not encountered in this unit, as shown in Fig. 11.

While Dover was a well-known early brickmaking center due to the ample clay resources in its surrounding tidal rivers, it was clear during excavation that the uncovered bricks did not date to the seventeenth century. These bricks are uniform in size. In the seventeenth century no regulations constrained brick mold size, meaning that seventeenth -century handmade bricks were never so regular and homogenous in form and composition (Garvin 1994). Brick production ramped up in Dover in the 1800s when demand increased for building projects. Over 30 brickyards operated along Dover Neck during the 1800s (Garvin 1994:29). These brickyards used standardized molds but employees still hand-molded the bricks. Hand-molded bricks from New Hampshire could not compete with more industrialized brick production that picked up across the United States starting in the late 1800s and early 1900s, however, so most of Dover’s brickyards stopped manufacturing by the turn of the twentieth century. The more industrial brick production of the twentieth century used higher firing temperatures and a wire-cut approach. These bricks’ purple hue indicates a higher firing temperature, while linear traces suggest wire-cutting remnant impacts (Fig. 10). Together, the aspects of these bricks suggest they were mass-manufactured bricks from the twentieth century.

This dating frame is further supported by the dominant artifact recovered in these units. Excavation produced numerous steel wire brad and wire nails. The nail industry transitioned from iron cut nails, to steel cut nails, to steel wire nails over the course of the 1880s to ca. 1900. Cut nail production peaked in 1886 and wire nails became more common thereafter, achieving predominance in the nail industry after 1897 (Adams 2002: 69-71). The numerous wire nails indicate this feature dates to the twentieth century, not the seventeenth century.

In addition to these findings, three 7/64 diameter bore hole kaolin pipe stems were recovered, dating to ca. 1650-80 (Harrington 1954). This indicates that while the brick feature did not date to the seventeenth century, it had intruded upon seventeenth-century archaeological materials. In other words, this much newer feature had disturbed aspects of the archaeological signature of the early colonial occupation of this site. Even at a protected site like this 1654 meetinghouse, the long-term continuous occupation of New England’s landscape poses challenges of intermixture, disturbance, and erasure that must be navigated in archaeological research (see Johnson and Ouimet 2018 for one multi-method approach to working in New England’s complexly layered landscapes).

While it was clear this brick feature was not an early colonial era feature and instead was a twentieth-century feature intruding on an early colonial site, GBAS’s extensive background research on this site produced no records indicating something of this later vintage should be present at the site. In deciding to explore this surprising find further, and to investigate other potential historical transformations and social processes, the GBAS project team recognized the sensitive nature of this site and did not want to expand exploratory excavations broadly. Given the bricks were relatively shallow, the project team decided to take chaining pins and crawl across the ground outwards from the original brick row, placing the pin in the ground every ca. 10 cm to see if it hit a possible brick. Through this method it became apparent that the bricks most likely continued to run linearly east-west. The team chased this row with this improvised non-destructive chaining pin approach until possible brick was no longer encountered, which occurred about 4 m (13.1 ft) east of the original unit.

**Fig. 12:**
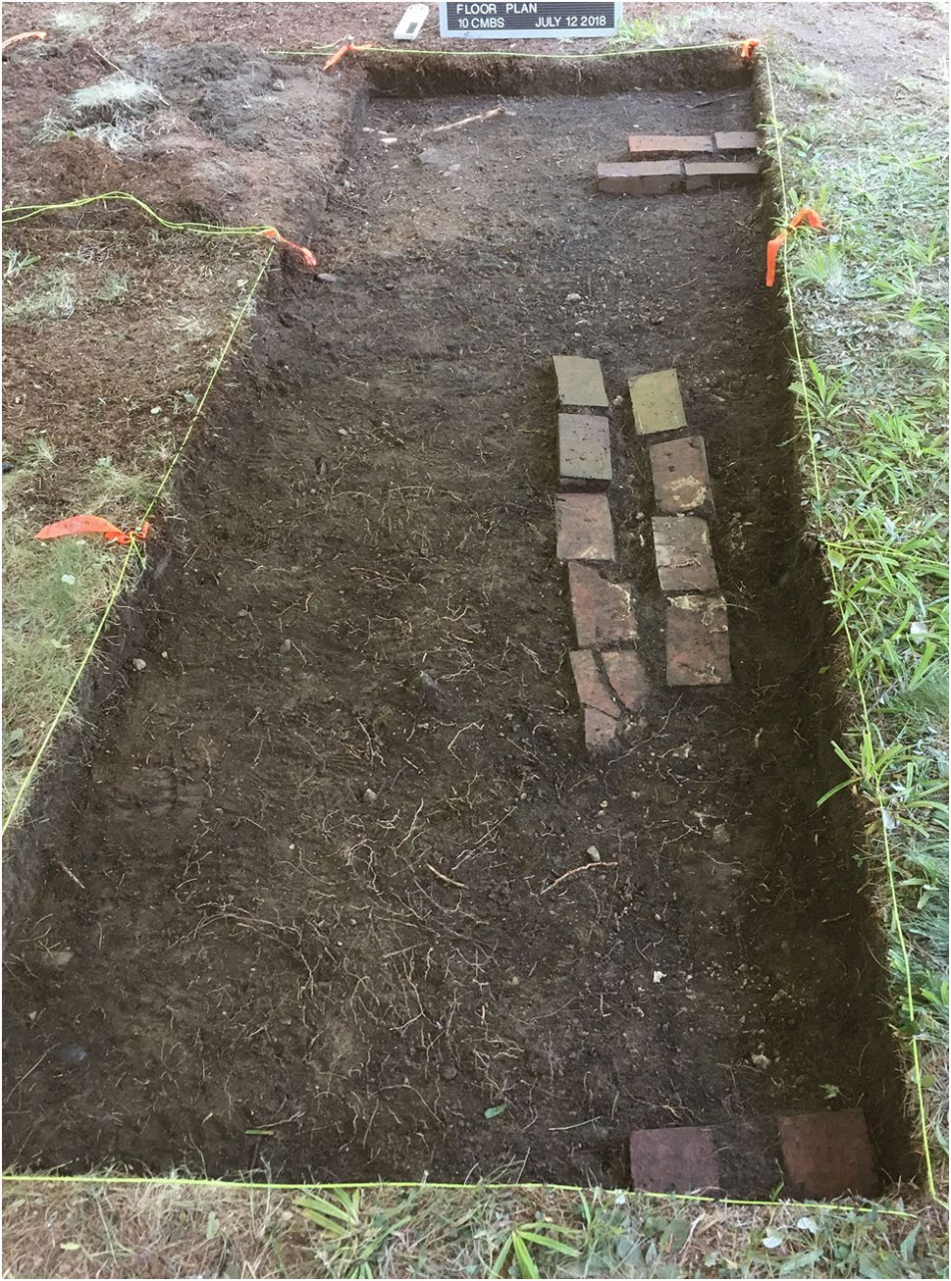
Two openings in brick feature (60 cm each; Units N 502.75 E 505.88, N 501.75 E 505.88, and 500.75 E 505.88) (photo by M. Howey, GBAS)

Accordingly, the project team opened a 1x1 m unit at N 502.75 E 505.88 (Fig. 9). The two-by-two row of bricks was found again situated in a relatively shallow manner in Level 1. The brick row protruded about 30 cm into the unit and stopped, which matched the chaining pin information that the brick feature ended in this vicinity (Fig. 12). No corner was found; the brick did not turn north-south at this terminus (Fig. 12). Again, the GPR indicated a rectangular anomaly that suggested there should be a continuation of the brick feature north-south. Chaining pin survey moved south from this end point and found the brick picked up again about 60 cm south. Another unit was opened here (N 501.75 E 505.88) and the two-by-two row of bricks was found again in Level 1. Bricks continued into the southern unit which was opened (N 500.75 E 505.88). Here excavations showed another gap in the row of bricks that was likewise about 60 cm and then the row of bricks picked up again at the far end of the unit (Fig. 12). At this point the project team stopped opening units. The original test unit was dug down below the brick to confirm it was only one brick deep and that there was no significant occupation below the brick.

The two openings found in the three units increased the sense that the row of bricks were placed to outline and/or support some kind of structural *replica* of the 1654 meeting house. The dimensions of the rectangular area found in the GPR results were strikingly close to the original dimensions of the meeting house at “forty foot longe, twenty six foot wide.” That description also indicated the presence of “two doors.” The two openings in the brick row are the same size, both 2 ft wide (60 cm), reflecting, possibly, two doors. While this one-deep, two by two row of bricks could not support a long-term, structurally sound construction, the fact that the spacing between the bricks was uniformly 5.5 cm everywhere adds to the sense that these bricks were placed to support some kind of temporary structural replication of the meeting house. This is because lumber is cut in standard thicknesses and an 8/4 cut is 2 and 1/8th inches (or 5.4 cm). The original order for the meeting house said to “plancke all the walls.” Numerous nails were recovered in all the units excavated around the bricks, as was thin windowpane glass. The nails and window glass further suggest a structure that was erected upon these bricks, and that there was more than just an outline of the meeting house dimensions crafted in this locale at some point in the twentieth century. The original meeting house specifications call for “glass and nails.”

Increasingly confident this was an early twentieth-century feature that was some kind of replica of the 1654 meeting house site, the GBAS project team began evaluating what specific activity most likely accounted for this feature. While analytic techniques like microstratigraphy might be productively employed to narrow down the brick feature’s dating in the future, without such analysis available currently, GBAS returned to documentary research. This process underscores the importance of working dynamically between material and archival-documentary streams of evidence, and using each to pose new questions and avenues of investigation. The last written record attesting to construction activity at the site stood strong with the DAR fence and stone wall erection in 1908. Records were found showing that in the 1910s some First Parish Church’s Sunday school teachers took students to visit the site, but there was no indication of associated construction activities. The small white sign that stands today was erected in the 1950s, but it is not located near these bricks.

### Glorifying while Destroying, and Broader Contexts for Memorial “Replicas”

Given these different forms of evidence, what is the most robust interpretation of this multilayered, multivalent finding? Reflecting upon what we know about the 1923 commemorative pageant, including its emphasis on direct historical connection to early English settlers; its valuation of landscape proximity to early colonial structures and terrain of significance; and its participants’ construction of other replicas for use in the performance of the spectacle, we interpret these finds as remnants of a replica of the 1654 meeting house built for the 1923 Tercentenary Pageant.

The dimensions, doors, nails, and glass all match the 1652 call for the new meeting house closely. We know pageant planners used historic documents to try to render this pageant historically accurate in terms of fidelity to original materials and locales. Another local newspaper article from the time emphasizes how, in order to make the pageant a success, historic episodes had been “unearthed in history books,” with the goal of having them “reproduced with the greatest possible truth to detail” (Tapley 1923). In addition to the town record laying out the specifics of the meeting house, pageant planners would have had access to the unsourced image of this meeting house (Fig. 6). While George Wadleigh wrote his *Notable events in the history of Dover, New Hampshire from the first settlement in 1623 to 1865* in 1882, he died before revising and publishing this work. In 1913 The Tufts College Press published it so that “his work may not be entirely lost” (Wadleigh 1913: Notice). This published image situated the entrance through the fortification into the site erroneously. It depicts it on the eastern wall, whereas GBAS’s survey of the site shows the likely gate/entrance through the fortification berm to be located on the south wall (Fig. 9). The location of this twentieth-century brick feature near the east wall suggests that it was built to be close to where the image in Fig. 6 shows the meetinghouse. That is, it seems the twentieth-century construction of this brick feature aimed to place it where the actual 1654 meeting house had stood. This aligns with wider pageant planning efforts at verisimilitude by seeking to assert authenticity through place.

Dover’s Tercentenary pageant gathered together disparate objects, histories, memories, places, and people, but it did not do so to cultivate new, more heterogenous relationships and futures. It did so with the aim of (re)telling a singular, and exclusionary, narrative of belonging. Dover’s pageant planners were placemakers, and placemakers can “selectively seek to cultivate certain responses, and therefore, attempt to define for others what should be remembered and how it should be remembered” (Rubertone 2008a:13). The planners enacted a normatively defined standard in colonial New England that “real” history commenced with English colonization (O’Brien 2010), and they publicly enforced a history controlled and applied by the socially and politically dominant: a “rightful” group of citizens, defined by genealogical connections between original English colonists and their descendants (Gorsline 2015: 293). Through assertions of proximity to seventeenth-century English colonialism in body, blood, and place, Dover’s Tercentenary spectacle marked 1623 as the firsting of “settlement,” deftly erasing Native presences, sovereign polities, and complexities of cross-cultural interactions. Moreover, it marked English colonial descendants as the lasting rightful citizens.

Ironically, even as pageant organizers strived to use geographic proximity of their replicas to glorify the English colonial founding of Dover, they simultaneously seem to have physically damaged at least one actual early colonial site--the 1654 First Parish Meeting House. The error in the location of the entrance in the available image of the meeting house appears to have been the reason the archaeological signature of the actual 1654 meeting house was left intact, which GBAS found in other GPR-led test excavations. Only an error saved the actual early colonial feature at the site from much more significant impacts. If the replica brick feature had been successfully located entirely where the actual meeting house stood, it would have succeeded in destroying a notably rare and significant site. It is also a place that holds material evidence of diverse lived experiences of early colonialism not captured in purist-oriented, exclusionary settler memoryscapes.

The material impacts caused by colonialist commemorative activities merit critical attention. These stagings did not unfold across blank, neutral, or unstoried landscapes, but instead took place within--and impacted--terrain already deeply transformed by natural and human processes. Their effects on the land contributed to existing palimpsests of cultural and social activities: layers upon layers of traces and meanings, some more legible than others, depending on the perspectives and values brought to bear upon these places. The Dover Tercentenary also presents considerations about commemorative activities as not merely additive but potentially also destructive, in ways that manifest alternate sensibilities about historical preservation and authenticity. The intended destruction of the original meeting house footprint was not iconoclastic, in the sense of deliberately defacing or eliminating an extant site or image for political-cultural-religious reasons, especially during times of contestation or regime change. Yet it nonetheless undermined the site.

Dover pageant organizers prioritized a highly visible enactment of memory that valorized particular claims to Anglo-American identities and belonging. They expended much less energy attempting to preserve the material integrity of the terrain’s stratigraphy in which they situated their program, even as they clearly valued the *surface* of the locale for its resonances of historical authenticity. These actions underscore the evolving nature of historical preservation sensibilities, and their variable forms even within Euro-colonial contexts, not to mention across cultural-social domains (Martinko 2020). From the vantage of the twenty-first century we bring other priorities and values, including recognition of how mid-seventeenth-century vernacular architectural traces can attest to English colonial ideas, behaviors, and relationships with the Native people of the Piscataqua region, all of whom lived and interacted in close proximity.

The re-emergence of the 1923 replica’s traces invites comparative reflections on the relative durability or ephemerality of New England memoryscapes. Colonial commemorative activities frequently left behind tangible artifacts in the landscape. Euro-colonial antiquarians installed heavy boulders across the Northeast terrain, inscribed with commemorative lettering or bedecked with bronze plaques that broadcast filiopietistic narratives of conquest, suffering, heroism, or other storylines that aimed to valorize and legitimize colonial claims to place and political authority. Many of these installations from the late nineteenth and early twentieth centuries persist in public spaces today, perpetuating colonial framings of the land and continuing the erasure of Native peoples and their own place-relations. Some monuments have been actively challenged or engaged by Native people themselves (see DeLucia 2018; O’Brien 2010; Rubertone 2008b, 2012), while others have been moved or gone missing (such as the “Canonicus” memorial boulder formerly situated in Providence, Rhode Island’s North Burial Ground; see Rubertone 2020).

**Fig. 13:**
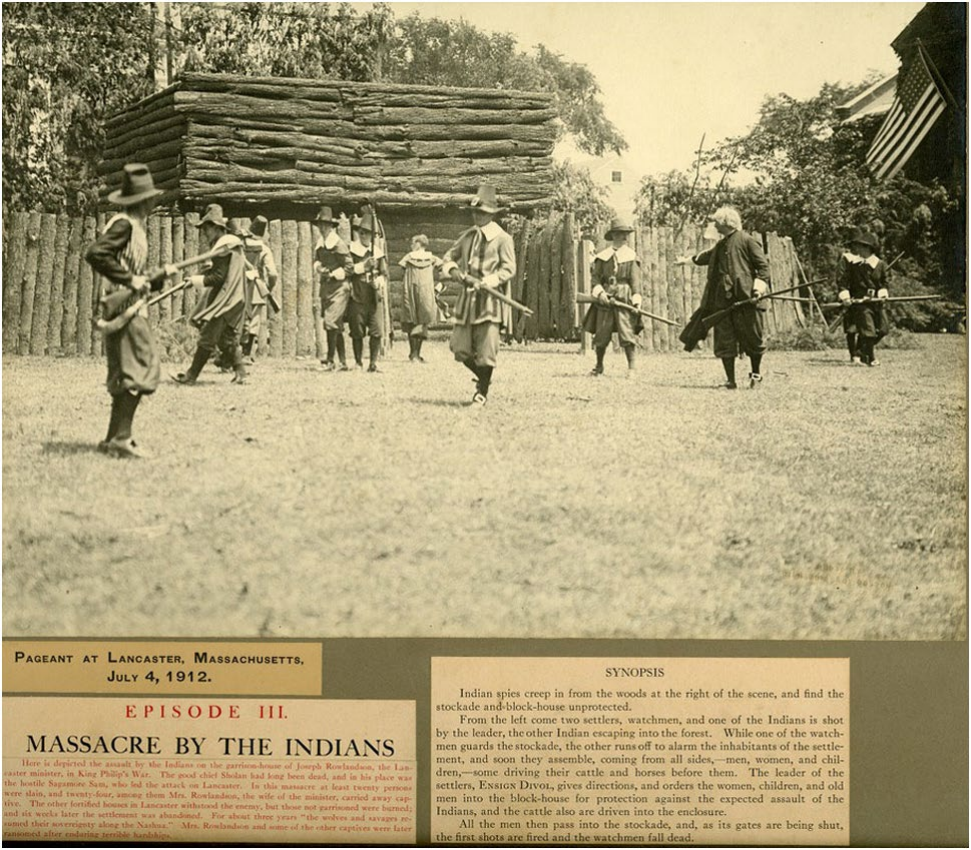
Photograph of the “Pageant at Lancaster” staged on the Fourth of July, 1912, reenacting Native attacks upon colonial Lancaster, Massachusetts, during the Indigenous resistance movement and colonial conflict often known as King Philip’s War (1676) (Panel 10). Courtesy of the Thayer Memorial Library of Lancaster, Massachusetts

**Fig. 14:**
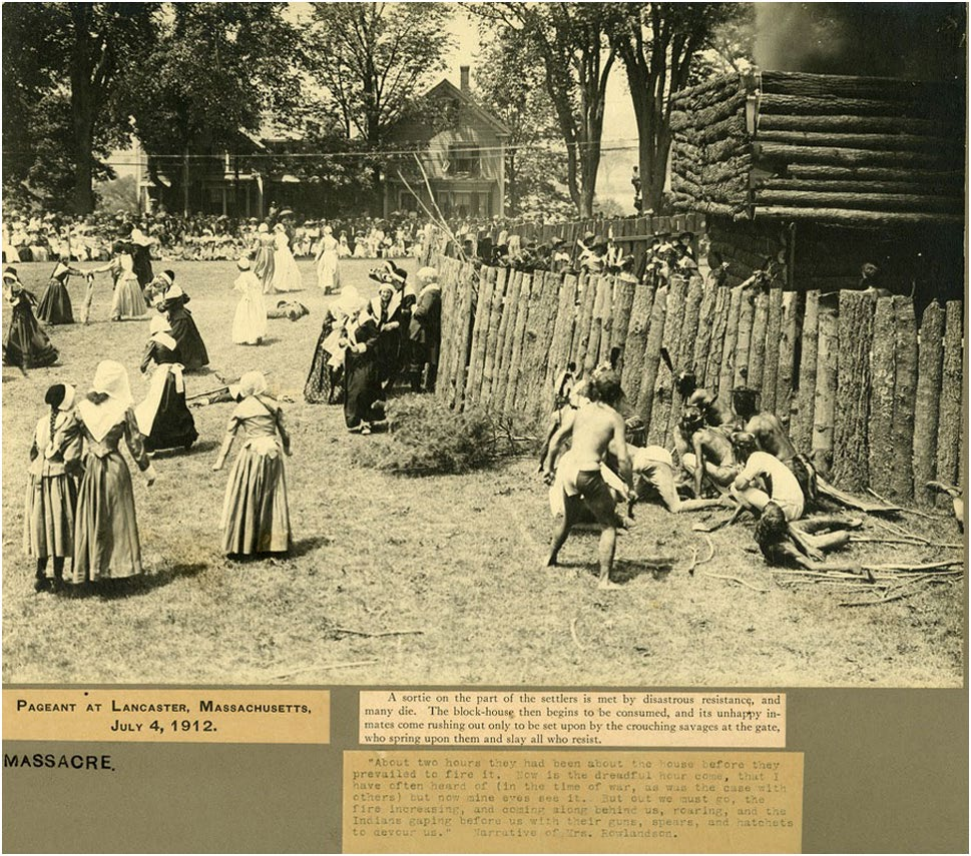
Photograph of the “Pageant at Lancaster” staged on the Fourth of July, 1912, reenacting Native attacks upon colonial Lancaster, Massachusetts, during the Indigenous resistance movement and colonial conflict often known as King Philip’s War (1676) (Panel 13) Here the blockhouse replica is being set on fire (top right). Courtesy of the Thayer Memorial Library of Lancaster, Massachusetts.

Less well recognized are more ephemeral structures, including those built from wood and other organic materials, that commemorative participants crafted in the course of their pageants and programming. To consider just one example, Euro-colonial organizers of the 1912 Fourth of July pageant in Lancaster, Massachusetts, built a replica wooden stockade that purported to resemble the late seventeenth-century fortifications in that town during King Philip’s War. This was a major multitribal resistance movement and colonial conflict (1675-78) that shifted regional relationships in enduring ways, and became a prominent touchstone for town identity in the ensuing centuries. In a pageant episode titled “Massacre by the Indians”--phrasing that cast Native people as the instigators of violence against colonial victims, rather than recognizing the structural violences wrought by settler colonialism upon Native populations--local residents of Lancaster re-enacted an attack by a coalition of Algonquian forces on Lancaster in early 1676. In this tumult Mary Rowlandson, the high-status wife of the town’s minister, and other colonists were taken captive, while others were wounded or killed (Brooks 2018). Photographs from the pageant document the elaborate replica “garrison house” or “blockhouse” of Reverend Joseph Rowlandson around which this colonialist theater transpired (Figures 13 and 14).

The replica provided a staging ground for performances of colonial martyrdom, as well as opportunities for local white residents to engage in racialized forms of “playing Indian,” to invoke Dakota historian Philip J. Deloria’s (1998) concept (see Fig. 1 as well). They used stereotypical, Plains-style costumes and body paint to signify generic, hostile “Indian-ness” in order to solidify their *own* identities as white Euro-American descendants of colonizers who had endured epic struggles to make homes in a new land--places that were, and still are, Nipmuc homelands (Pezzarossi et al. 2021). The faux-military actions and smoke that transpired in the production framed violence as entertaining theater referencing a distant past, not an ongoing condition of twentieth century life. Moreover, by staging this event on the Fourth of July, pageant organizers linked ideas of US independence to Native/colonial conflicts, marshalling Lancaster colonists’ experiences in 1676 into a larger arc of American national emergence.

Attendees of the spectacle inscribed further layers of meaning by photographing and publicizing the pageant, codifying it as a touchstone of local memory through archiving and print culture dissemination (Fig. 13). This visual record-keeping was an essential counterpart to the transience of the blockhouse itself, which was set on fire at a dramatic culmination in the pageant (Fig. 14). In recent years, the Thayer Memorial Library in Lancaster, a public institution founded in 1868, has digitized many of its Special Collections materials, including photographs of the pageant. By archiving and disseminating these primary sources in new ways, the potential uses and meanings of these representations may continue to transform as different questions and critical perspectives are brought to bear on them.

**Fig. 15:**
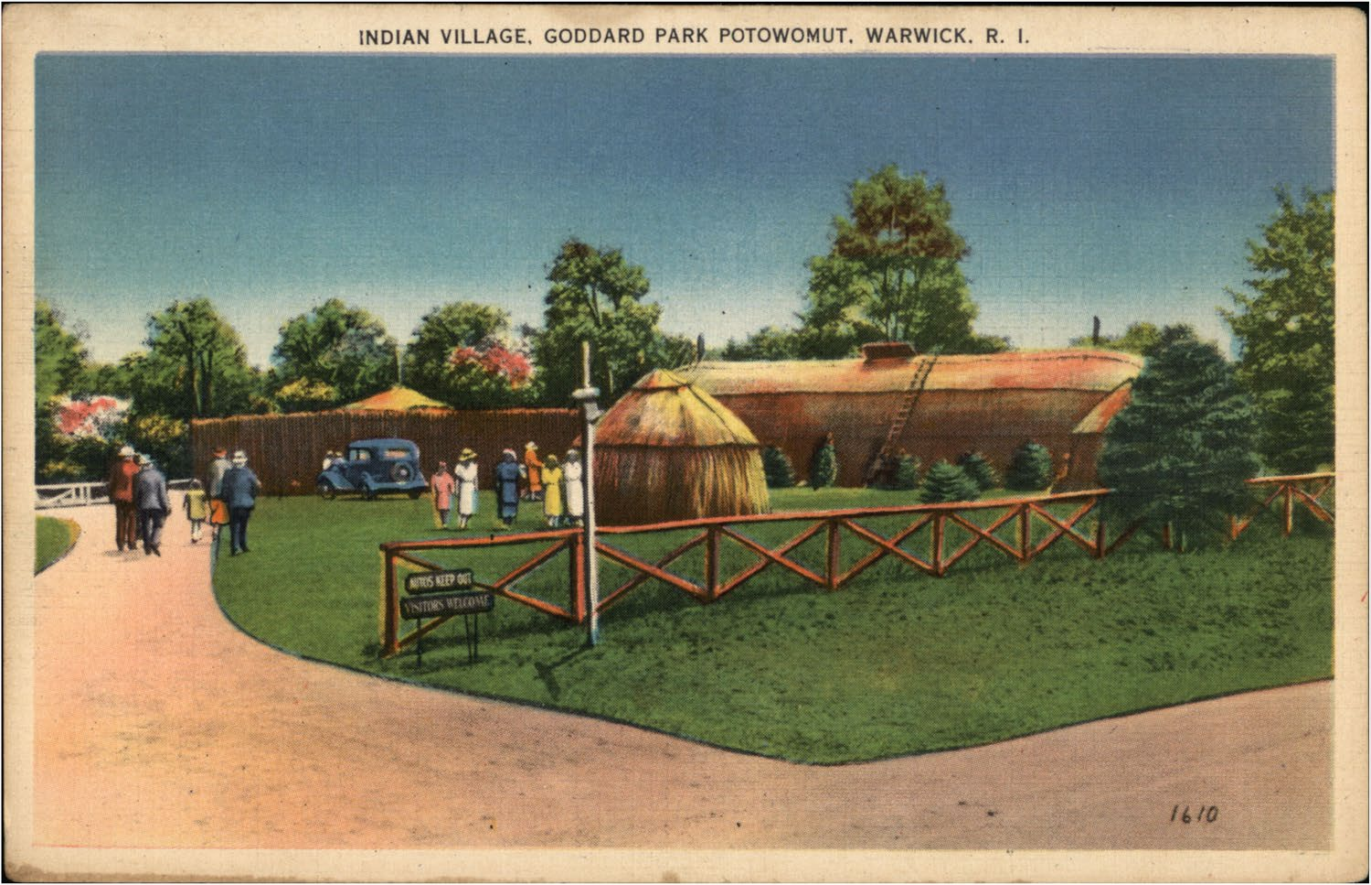
“Indian Village” at Goddard Park in Warwick, Rhode Island, erected during the Tercentenary of the colonial founding of Rhode Island, and operated in conjunction with the first annual “Indian Day” supported by Narragansett and other regional tribal people. Image from CardCow.com

Not every replica or material recreation reinforced colonialist memory production. Native people themselves at times mobilized these forms to advocate for very different purposes, transforming “replication” into a critical site of contestation and counter-memory. In 1936 members of the Narragansett Tribe took leading roles envisioning, designing, and constructing an “Indian Village” at Goddard Park on the west side of Narragansett Bay (Fig. 15). Located at Potowomut, a place of longstanding Indigenous significance in the fertile coastal region, the village was created in the course of a Works Progress Administration (WPA) project during the Great Depression, undergirding it with both labor and civic significance during an era of pronounced social and economic stress. The village formed an integral part of “Indian Day” celebrations associated with the Tercentenary commemoration of the founding of Rhode Island as a colony in 1636. Narragansett people strategically used this newly built constellation of vernacular architecture to emphasize and enact the continuance of Narragansett people, identity, and culture in the twentieth century. They pushed back against colonial narratives, laws, and policies that had presumed to spell the termination of Narragansett presences and tribal sovereignty, particularly through the illegal “detribalization” enacted by the State of Rhode Island in the 1880s (Rubertone 2008b).

Featuring a longhouse and other buildings constructed using traditional materials and techniques, the village was enough a point of community pride that a lengthy write-up about it featured in *The Narragansett Dawn* (1936), a tribal magazine published by Princess Red Wing in the mid-1930s (First Indian Day) (Fig. 15). The village’s opening ceremony featured a multitribal gathering and remarks from Wampanoag Chief Ousa Mequin/Yellow Feather (Rev. Leroy Perry), minister at the Native church at Gay Head/Aquinnah, as well as other participants. The *Dawn*’s coverage emphasized the historical accuracy of the village. It acknowledged extensive research and advisory input by non-Native archaeologists at Harvard University and Phillips Academy, Andover in order to make it “designed in accordance with the best available records of the Colonial period.” One concession to the changing times--such as environmental degradation of forests, a direct effect of extractive colonialist land use--was the use of artificial bark to cover the structures “because of the scarcity of the bark of the required size and a desire to not cut what little is still growing” (First Indian Day). The 1936 Indian Village was a vibrant Narragansett and intertribal gathering site: a place for socializing, planting in the community garden, and using intentional self-representation to educate non-Native visitors about Native, particularly Narragansett, resilience and resistance.

Narragansetts were highly attuned to the wider pageantry phenomenon and transformed the genre to serve their own purposes by staging dramatic performances such as “The Coming of Roger Williams to the Lodge of Canonicus,” held at Camp Ki-Yi as part of the Rhode Island Tercentenary. This event, perhaps intentionally held on July 4-5 as a *counterpoint* to celebrations of the US nation’s founding, enabled Narragansett and other tribal people to assume leading roles in portraying early Native-colonial encounters from tribally centered perspectives. Its program appeared in the *Narragansett Dawn* (1936) and attested to an extensive cast of tribal members, who performed episodes that moved well beyond seventeenth- and eighteenth-century histories to span right up to the present day. As Christine Reiser (2009) argued in her study of Northeastern Native involvement in pageantry in early twentieth-century southern New England, “Refusing to be consigned to the past, New England Native American groups re-authored public historical imagery in demonstration of their abilities to be both Indigenous and modern citizens.” Reiser focused on Native people’s use of regalia, bodily adornment, and other forms of expression as active modes of remaking and solidifying group identities in the face of erasive and reductive colonial expectations that relegated Native people to a distant, monolithic past. Her argument might be productively extended to placemaking and attendant uses of vernacular architecture to re-signify in meaningful geographies. The “Indian Village” deployed “tradition” in complex ways: rather than constructing homes of the kind that many Narragansett people inhabited in the mid-1930s (e.g., timber-frame houses and multistory urban apartment buildings with running water and electricity), participants instead hearkened back to ancestral architectures such as bark- or mat-covered sapling frames comprising *wetuash*. They may have been savvily recognizing non-Natives’ expectations about what “authentic” Narragansett practices entailed, then using this site to speak directly to visitors about their own experiences of Indigenous modernity and commitments to the endurance of community sovereignty.

Indeed, the “Indian Village” may help complicate conceptualizations of “replicas” and their associated temporalities. On one level Native builders were recreating older architectural forms. Yet on another they were continuing or reanimating ancestral forms of knowledge and craftsmanship, carrying forward practices that had been severely disrupted by settler colonialism. In this latter respect the term “replica,” in the sense of reproduction or mimicry, seems inadequate to the multilayered significances of this embodied, material type of engagement with the past. These more complicated dynamics anticipate the transhistorical dimensions of how Native people in the twenty-first century, such as workers at the Wampanoag Indigenous Program (WIP) at Patuxet-Plimoth Plantation, a “bicultural institution,” have engaged in extensive (re)production of Wampanoag material culture and architecture (Coombs 2014). As Lisa King (2019) has written about the WIP, “the people in the Wampanoag Homesite are actual Indigenous people who are not treating the lifeways they interpret for visitors as ‘past,’ but rather ongoing meaning-making from a Wampanoag point of view.” 

In the course of its construction and use, the “Indian Village,” like the Dover replica meetinghouse, may have disrupted underlying historic-ancestral taskscapes of cultural significance, though for Indigenous- rather than colonialist-led reasons. The “Indian Village” structures seem to have been dismantled shortly after the conclusion of the commemoration. Today visitors to Goddard State Park encounter no visible traces of their presence, nor public testimonies about the significant crosscultural and intertribal events that transpired there in 1936, though the village may well remain a place recollected in community stories as well as accessible through archival documentation. There is also a surprising ephemerality to the park’s colonial memoryscape. The Tercentenary commemoration encompassed the dedication of a 9,500 lb (4,309 kg) memorial boulder honoring Captain Michael Peirce, a colonial militia leader killed by Narragansetts during King Philip’s War. A descendant of Peirce dedicated the massive stone memorial, akin to the situation in Dover of colonial “blood” and genealogy being deployed as modes of authentication. This boulder comprised just one piece of an extensive commemorative landscape focused on Peirce. Yet the plaque affixed to it was later stolen and few people today, including Peirce family members, even recall the origins of the monument (LaCroix 2011).

The Narragansett “Indian Village,” sited in southern New England, offers a striking point of contrast to the commemorative forms and processes in Dover. In that latter, more northern area, no such highly visible counter-commemorations by Native people seem to have occurred in 1923 or in other proximate moments. Viewed comparatively, the intensely monovocal quality of the Dover pageant stands out for its strongly colonialist mode of engaging the seventeenth century. Explaining the apparent absence of publicly visible Native counter-memory in a full manner would exceed the bounds of the present paper, but divergent histories of Indigenous resistance, forced removal and colonization, and strategic under-visibility for purposes of survival may have shaped the variable outcomes. Native people from multiple tribal communities *did* continue to inhabit the Piscataqua River and seacoast region, and they participated in place-making and mobility in ways that supported their livelihoods and kinship networks. Owing to the severe multicentury impacts of colonialism and territorial dispossession, however, no formal tribal reservations existed in this area, and the absence of such defined community spaces may have affected the dynamics of local commemorative activities (DeLucia 2012).

Today communities across the Northeast are reckoning with the 400th anniversary of English settler colonialism’s arrival in the region, engaging in discourses and practices that range from multicultural inclusion to more forcefully decolonial and Indigenous-centered approaches. The most prominent has been the “Plymouth 400” events in 2020, marking 400 years of English presence in Wampanoag homelands. That commemoration, while transformed from in-person to primarily virtual gatherings owing to COVID-19, exemplified shifting discursive and social terrain, owing largely to the concerted efforts of Wampanoag culture-keepers and program organizers. In contrast to the 1920 commemoration and its Pilgrimcentric celebrations (Handsman 2008), 2020 much more extensively focused on Wampanoag and other Native memories, forms of knowledge, and critical perspectives on colonization’s traumatic effects (Blee and O’Brien 2019, 2021; Wisecup 2021). Yet the Plymouth spectacle also presented challenges about how colonial experiences and narratives continued to be centered, as opposed to enacting fuller decolonial transformations prioritizing long-standing Wampanoag continuities, voices, and sovereignties. Additionally, the commercialization of such commemorations--partly intended to drive tourist traffic and generate revenue for heritage organizations--may present difficulties in frankly reckoning with violence, dispossession, genocide, enslavement, and other forces that have profoundly impacted Native communities and tribal nations into the twenty-first century.

Dover has already launched its Quatercentenary commemoration with a virtual speaker series leading up to in-person events in 2023 if public health conditions permit. As this commemoration takes shape, it is important to consider how it will differ from the memorial practices of 1923. Will it grapple with narratives and memoryscapes of whiteness, colonization, and dispossession in the region? What anxieties of our time might be addressed and expressed in these celebrations--or downplayed, even negated? How can we remain mindful of the impacts of formally acknowledging such constructed “beginnings,” and the forms of historical periodization and value that they entail? If architectural “replicas” are proposed, will planners conscientiously seek input from (and meaningfully respond to) multiple stakeholders, including Indigenous representatives and archaeologists, about the impacts such activities might have on sensitive cultural landscapes? It remains to be seen whether these commemorations reinscribe the problematic power relations of other regional public history sites, where Siobhan Hart (2019) has argued that “projects place the burden of decolonizing squarely on Indian people, expecting little of non-Indians except open minds and a willingness to consider other points of view.” Or whether their organizers and participants take up the challenges of epistemological transformation, relationship-building, and authority-sharing that have begun to cohere in other parts of the region, such as Nipmuc homelands (Gould et al. 2020).

The Dover memorial activities are indeed off to a different start than in 1923. The speaker series opened with a land recognition from local Indigenous leader, Kathleen Blake, Chair of the New Hampshire Commission on Native American Affairs, and the second talk was given by regional Indigenous leaders Paul W. Pouliot, Sag8mo, and Denise K. Pouliot, Sag8mosquaw (head speakers) of the Cowasuck Band of the Pennacook Abenaki People (their talks are viewable here: https://www.dover400.org/). Their presences, voices, and critical interventions into celebratory colonial narratives attest to Indigenous continuance in this region despite the ruptures caused by colonialism. The work of local tribal leaders, as well as leaders from other marginalized communities, especially African Americans, press commemorative organizers to proceed differently in these twenty-first century reckonings with the past. They offer opportunities to layer memory and materiality in more inclusive ways, and to transform structures of power into other ways of gathering and belonging.
